# TAZ Is Related to Postoperative In-Hospital Mortality of Acute Type A Aortic Dissection

**DOI:** 10.3389/fcvm.2020.587996

**Published:** 2020-11-03

**Authors:** Wenjian Jiang, Yuan Xue, Haibin Li, Hongjia Zhang, Yuanfei Zhao

**Affiliations:** ^1^Department of Cardiac Surgery, Beijing Anzhen Hospital, Capital Medical University, Beijing, China; ^2^Beijing Institute of Heart, Lung and Blood Vessel Diseases, Beijing, China; ^3^Beijing Lab for Cardiovascular Precision Medicine, Beijing, China; ^4^Beijing Chaoyang Hospital, Capital Medical University, Beijing, China; ^5^Centre for Transplant and Renal Research, The Westmead Institute for Medical Research, University of Sydney, Sydney, NSW, Australia

**Keywords:** aortic dissection, heart surgery, mortality, ascending aorta and total aortic arch replacement, TAZ

## Abstract

**Background:** Surgical repair of acute type A aortic dissection (ATAAD) has high risk and mortality, and there are few biomarkers of postoperative in-hospital mortality until now. This study investigated the association between WW domain–containing transcription regulator protein 1 (TAZ) and the postoperative in-hospital mortality of ATAAD patients.

**Methods:** This is a retrospective cohort study. Data and blood samples were collected from 95 consecutive patients with ATAAD who underwent surgeries in our hospital from July 1, 2016, to December 31, 2016. The data collection included all the risk factors introduced by the modified EuroSCORE (European System for Cardiac Operative Risk Evaluation). The predictors of postoperative in-hospital death were confirmed by univariate regression analysis. Multivariable logistic regressions were used to analyze the association of the preoperative plasma level of TAZ and the postoperative in-hospital mortality of ATAAD patients. In addition, we used the generalized additive model to identify non-linear relationships.

**Results:** Three models were used in the multivariable logistic regression analysis of the relationship between the preoperative plasma level of TAZ and postoperative in-hospital death. In the crude model, the preoperative plasma level of TAZ showed a positive correlation with postoperative in-hospital death [odds ratio (OR) = 1.33, 95% confidence interval (CI): 1.01–1.74, *P* = 0.04]. In adjusted model I and adjusted model II, similar results were found (OR = 1.35, 95% CI: 1.01–1.80, *P* = 0.04 and OR = 1.35, 95% CI: 1.01–1.81, *P* = 0.04). The risk of postoperative in-hospital death in the preoperative plasma level of the TAZ≥12.70 ng/mL group was 10.08 times (OR = 10.08, 95% CI: 1.63–62.37; *P* = 0.01) that of the preoperative plasma level of the TAZ <12.70 ng/mL group.

**Conclusions:** The high preoperative plasma level of TAZ suggested poor surgical prognosis for ATAAD patients. The patients with a preoperative plasma level of TAZ ≥ 12.7 ng/ml had much higher postoperative in-hospital mortality.

## Introduction

Aortic dissection (AD), especially the acute type A aortic dissection (ATAAD) with dissected ascending aorta, is the most life-threatening vascular disease (1, 2). Based on the present guidelines, all patients with ATAAD should be transferred to the operating room if possible (3, 4). Although there has been a significant decline in the in-hospital surgical mortality rate of patients presenting with ATAAD with the advancement of related technology, surgical repair remains high risk and has a high mortality rate (3.09–30.00%), which deters most aortic surgeons (2, 5, 6). Some preoperative presentations, such as malperfusion phenomena, are considered predictive factors for postoperative mortality (2). However, there are few biomarkers of postoperative mortality until now.

WW domain–containing transcription regulator protein 1 (TAZ) is ubiquitous in the human body and mainly found downstream of the Hippo pathway, which regulates many fundamental biological processes (7). Our previous research focused on the molecular pathogenesis of ATAAD and found that altered mechanical stress induced the change of Hippo pathway, which contributes to ATAAD development (8). As TAZ play important roles in the development of ATAAD, they might also be related to postoperative mortality of ATAAD.

In this study, we investigated the preoperative plasma levels of TAZ of patients with ATAAD and studied the association between the preoperative plasma level of TAZ and the postoperative mortality of ATAAD patients.

## Materials and Methods

### Patient Selection and Blood Sample Collection

**Figure 1:**
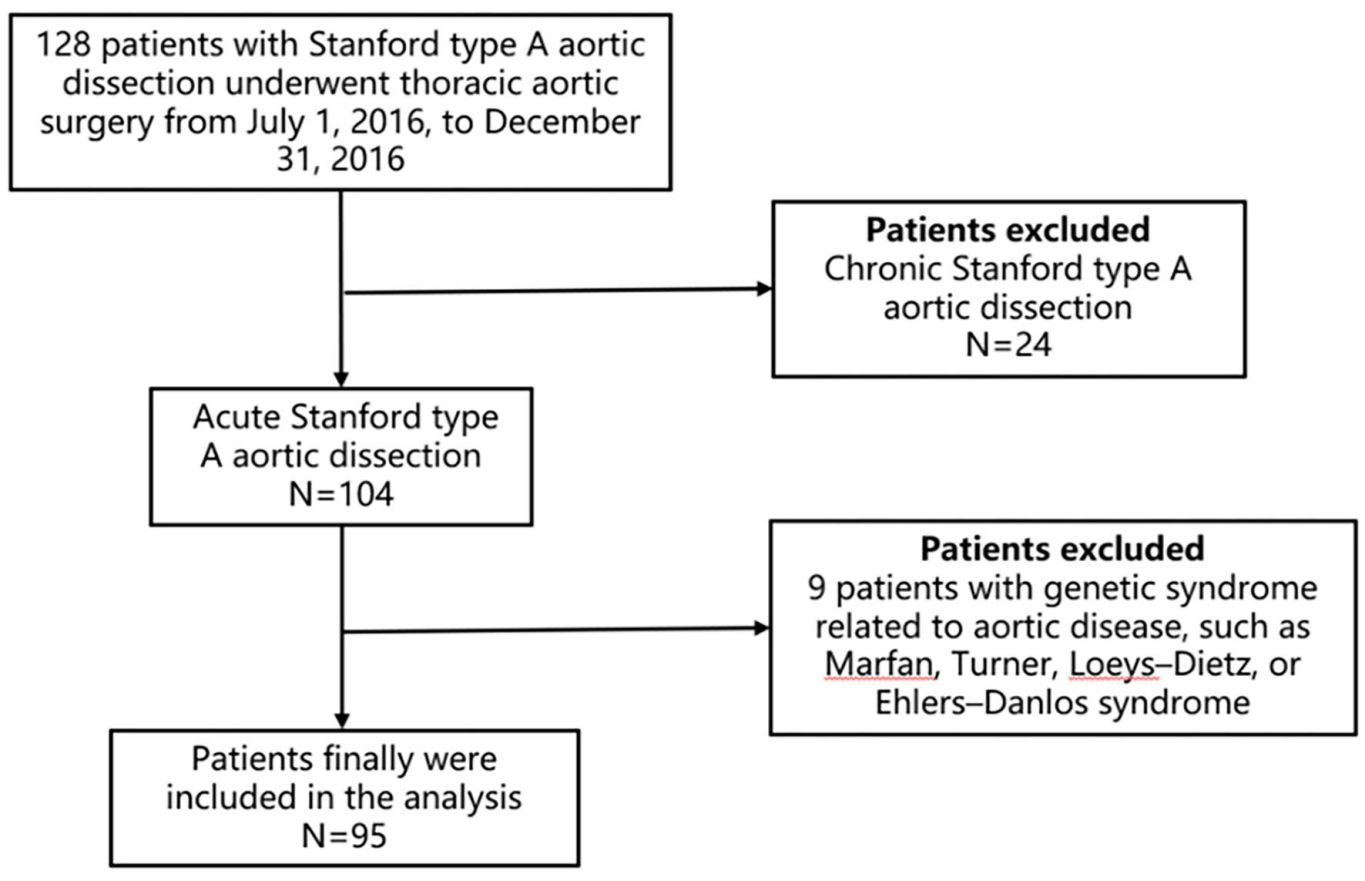
Flowchart of the screening and enrollment of study patients.

The human study and the use of human blood were approved by the Ethics Committee of Beijing Anzhen Hospital (Institutional Review Board File 2014019) and were consistent with the principles outlined in the Declaration of Helsinki. All patients (104) with ATAAD who underwent surgeries in our hospital and enrolled in “*A study of the prediction and the treatment of Acute Aortic Syndrome (ChiCTR1900022637)*” from July 1, 2016, to December 31, 2016, were included for the purpose of this analysis. After excluding nine patients with genetic syndrome related to aortic disease, such as Marfan, Turner, Loeys–Dietz, or Ehlers–Danlos syndrome, 95 consecutive patients were involved in the final analysis (Figure 1). All the patients received the surgery in 24 h after the onset of the disease. The surgical procedures included 67 patients with Bentall [one patient with CABG (coronary artery bypass grafting), one patient with MVP (mitral valvuloplasty) ± TVP (tricuspid valvuloplasty), 57 patients with total arch replacement using a tetrafurcate graft and stented elephant trunk implantation (5), one patient with total arch replacement using a tetrafurcate graft and stented elephant trunk implantation + CABG], 22 patients with ascending aortic replacement (one patient with CABG, 19 patients with total arch replacement using a tetrafurcate graft and stented elephant trunk implantation), and six patients with redo-operation (one patient with ascending aortic replacement, one patient with ascending aortic replacement + CABG, four patients with Bentall + total arch replacement using a tetrafurcate graft and stented elephant trunk implantation). The right axillary artery was used for antegrade selective cerebral perfusion when performing total arch replacement using a tetrafurcate graft and stented elephant trunk implantation under deep hypothermia circulation arrest.

All the patients received the surgery in 24 h after the onset of aortic dissection, and blood samples were collected from these patients before the surgical intervention was initiated.

### Blood Sample Assays

After collection, the blood sample was anticoagulated with sodium citrate and then centrifuged for 15 min at 3,500 rpm at 4°C. All the samples were divided into 0.5-ml per centrifuge tubes, stored at−80°C, and only used for this study. The mean sample storage duration was 84.50 days when ELISA was performed. The preoperative plasma levels of TAZ were determined by ELISA (Uscn, Wuhan, China), as previously described (9). All ELISAs were performed three times, and the mean value was used for analysis.

### Data Collection

Data were assembled from the database of “*A study of the prediction and treatment of Acute Aortic Syndrome (ChiCTR1900022637)*,” which was established based on standardized electronic medical records from our hospital. The data collection included all the risk factors introduced by the modified EuroSCORE (10), such as preoperative variables [age, sex, BMI (calculated based on height and weight), hypertension, diabetes status, pulmonary disease, extracardiac arteriopathy, neurological or musculoskeletal dysfunction, recent myocardial infarction, previous cardiac surgery, cardiac tamponade, left ventricular ejection fraction (LVEF), serum creatinine, critical preoperative state, unstable angina, d-dimer, platelet, fibrinogen, c-reactive protein, troponin I, ascending aorta diameter, pleural effusion, pericardial effusion, coronary arteries involved, location of intimal defect] and intraoperative variables (cardiopulmonary bypass time, aortic cross-clamp time and antegrade cerebral perfusion) and postoperative outcome variable (in-hospital death).

### Statistical Analysis

Data were expressed as frequency and percentage, as mean ± standard deviation (SD), or as median and interquartile range (IQR). *T* tests and Mann–Whitney *U* tests were applied according to the different distributions of the variables. Logistic regression analysis was performed to identify the predictors of in-hospital mortality. According to the recommendation of the STROBE statement, both non-adjusted and adjusted multiple regression models were used to evaluate the effect of the predictor on mortality after surgery. We selected these confounders on the basis of their associations with the outcomes of interest or a change in effect estimate of more than 10% (11), which included BMI (as a continuous variable) and cardiac tamponade (as a categorical variables). Moreover, the decision to add each measured potential confounder in the model was based on previous scientific evidence, namely, for cardiopulmonary bypass time (as a continuous variable), serum creatinine (as a continuous variable), and recent myocardial infarction (as a categorical variable). To explore multicollinearity among different covariates in the model, we excluded variables with variance inflation factor >5. In addition, the generalized additive model (GAM) was used to identify non-linear relationships. Receiver operating characteristic (ROC) curves constructed using Bootstrap resampling (times = 500) were used to find the optimal cutoff values for the preoperative plasma level of TAZ (with maximizing the sum of sensitivity and specificity) to predict in-hospital mortality. *P* < 0.05 was considered statistically significant (two-sided). All analyses were completed by the statistical software packages R (http://www.R-project.org, The R Foundation) and EmpowerStats (http://www.empowerstats.com, X & Y Solutions, Inc., Boston, MA).

## Results

### Baseline Characteristics of Participants

Table 1 lists the baseline characteristics of the 95 consecutive patients in this cohort. Nine patients (9.47%) died in the hospital [cardiogenic shock, low cardiac output syndrome (*n* = 1); septic shock (*n* = 1); lung infection, respiratory failure (*n* = 1), renal failure, paraplegia, heart failure (*n* = 1); cerebral infarction (*n* = 2); multiple-organ failure, electrolyte disorder, anemia (*n* = 1); cardiogenic shock, ventricular fibrillation (*n* = 1); distal dissection rupture, tachycardia (*n* = 1)]. The average preoperative plasma level of TAZ of these individuals was 11.76 ± 3.00 ng/ml. Compared with the surviving patients (11.55 ± 2.98 ng/ml), dead patients had a significantly higher preoperative plasma level of TAZ (13.80 ± 2.45 ng/ml) (*P* = 0.03).

**Table 1:** Baseline characteristics of participants in ATAAD.

**Postoperative in-hospital death**	**No**	**Yes**	***P*-value**
N	86	9	
TAZ, ng/mL, mean ± SD	11.55 ± 2.98	13.80 ± 2.45	0.03^*^
Age, years, mean ± SD	51.42 ± 11.67	47.11± 10.68	0.36
Sex (male) *n* (%)	58 (67.44%)	8 (88.89%)	0.27
BMI, kg/cm^2^, mean ± SD	25.69 ± 3.42	26.66 ± 5.51	0.69
Systolic blood pressure, mmHg, mean ± SD	126.07 ± 19.86	119.56 ± 19.12	0.28
Diastolic blood pressure, mmHg, mean ± SD	73.52 ± 11.10	73.56 ± 17.02	0.89
Heart rate, mean ± SD	81.00 ± 9.29	81.56 ± 7.32	0.46
Hypertension *n* (%)	42 (48.84%)	5 (55.56%)	0.70
Smoking history *n* (%)	25 (29.07%)	3 (33.33%)	0.72
Diabetes status *n* (%)	0 (0.00%)	0 (0.00%)	>0.99
Pulmonary disease *n* (%)	0 (0.00%)	0 (0.00%)	>0.99
Extracardiac arteriopathy *n* (%)	5 (5.81%)	0 (0.00%)	>0.99
Neurological or musculoskeletal dysfunction *n* (%)	2 (2.33%)	0 (0.00%)	>0.99
Previous cardiac surgery *n* (%)	11 (12.79%)	1 (11.1%)	>0.99
Recent myocardial infarct *n* (%)	58 (67.44%)	6 (66.67%)	0.96
Cardiac tamponade *n* (%)	8 (9.30%)	1 (11.11%)	>0.99
LVEF (%)	59.91 ± 7.85	62.89 ± 6.74	0.30
Serum creatinine, μmol/L, mean ± SD	84.48 ± 41.11	84.48 ± 41.11	0.53
Critical preoperative state *n* (%)	66 (76.74%)	8 (88.89%)	0.68
Unstable angina *n* (%)	3 (3.49%)	1 (11.11%)	0.28
D-dimer, median IQR, ng/mL	2051 (1006.25–3809.25)	3,341 (2,006–4,996)	0.26
Platelet, G/L, mean ± SD	154.45 ± 56.60	152.33 ± 37.92	0.91
Fibrinogen, g/L, mean ± SD	3.37 ± 1.71	3.08 ± 0.92	0.62
C-reactive protein, mg/L, mean ± SD	74.38 ± 27.66	78.24 ± 29.97	0.69
Troponin I, median IQR, ng/mL	0.08 (0.01-0.80)	0.04 (0.02–1.36)	0.51
Ascending aorta diameter, cm, mean ± SD	4.81 ± 0.73	4.81 ± 0.63	0.99
Pleural effusion **n** (%)	17 (19.77%)	2 (22.22%)	0.86
Pericardial effusion *n* (%)	21 (24.42%)	2 (22.22%)	0.88
Coronary arteries involved *n* (%)	5 (5.81%)	1 (11.11%)	0.53
Location of intimal defect *n* (%)			0.74
Ascending aorta	51 (59.30%)	6 (66.67%)	
Aorta arch	30 (34.88%)	3 (33.33%)	
Distal ascending aorta	5 (5.81%)	0 (0.00%)	
Cross-clamp time, min, mean ± SD	101.48 ± 41.54	105.22 ± 52.93	0.85
Cardiopulmonary bypass time, min, mean ± SD	187.87 ± 73.53	200.33 ± 53.94	0.29

*Results are expressed as n (%) or mean ± standard deviation (SD) or median interquartile range (IQR)*.

*BMI: body mass index; LVEF: left ventricular ejection faction; TAZ: WW domain –containing transcription regulator protein 1*.

* *P value indicates significance at P < 0.05*.

### Univariate Analysis of Predictors for Postoperative In-Hospital Death

The results of univariate analysis showed that the preoperative plasma level of TAZ was correlated with more postoperative in-hospital death. We also found that age, gender, BMI, systolic blood pressure, diastolic blood pressure, heart rate, hypertension, smoking history, recent myocardial infarction, previous cardiac surgery, cardiac tamponade, LVEF, serum creatinine, critical preoperative state, unstable angina, d-dimer, platelet, fibrinogen, c-reactive protein, troponin I, ascending aorta diameter, pleural effusion, pericardial effusion, coronary arteries involved, cardiopulmonary bypass time, and cross-clamp time were not associated with postoperative in-hospital death in this cohort (Table 2).

**Table 2:** The unadjusted association of clinical characteristics with in-hospital mortality (*N* = 95).

**Variables**	**Statistics**	**OR (95% CI)**	***P*-value**
TAZ, ng/mL, mean ± SD	11.76 ± 3.00	1.33 (1.01, 1.74)	0.04^*^
Age, years, mean ± SD	51.01 ± 11.59	0.97 (0.91, 1.03)	0.29
Sex (male), *n* (%)	66 (69.5%)	3.86 (0.46, 32.41)	0.21
BMI, kg/cm^2^, mean ± SD	25.8 ± 3.6	1.08 (0.89, 1.29)	0.45
Systolic blood pressure, mmHg	125.5 ± 19.8	0.98 (0.95, 1.02)	0.35
Diastolic blood pressure, mmHg	73.53 ± 11.66	1.00 (0.94, 1.06)	0.99
Heart rate, mean ± SD	81.05 ± 9.09	1.01 (0.93, 1.09)	0.86
Hypertension, *n* (%)	47 (49.47%)	1.31 (0.33, 5.21)	0.70
Smoking history, *n* (%)	28 (29.47%)	1.22 (0.28, 5.26)	0.79
Recent myocardial infarction *n* (%)	64 (67.37%)	0.97 (0.22, 4.15)	0.96
Previous cardiac surgery, *n* (%)	12 (12.63%)	0.85 (0.10, 7.49)	0.89
Cardiac tamponade, *n* (%)	9 (9.47%)	1.22 (0.13, 11.03)	0.86
LVEF, %, mean ± SD	60.22 ± 7.76	1.06 (0.96, 1.17)	0.27
Serum creatinine, μmol/L, mean ± SD	85.55 ± 42.72	1.01 (0.99, 1.02)	0.46
Critical preoperative state, *n* (%)	74 (77.89%)	2.42 (0.29, 20.57)	0.42
Unstable angina, *n* (%)	4 (4.21%)	3.46 (0.32, 37.24)	0.31
D-dimer, median IQR, ng/mL	2058 (1030.5–4252)	1.00 (1.00,1.00)	0.28
Platelet, G/L, mean ± SD	154.25 ± 54.95	1.00 (0.99, 1.01)	0.91
Fibrinogen, g/L, mean ± SD	3.35 ± 1.65	0.89 (0.58, 1.38)	0.61
C-reactive protein, mg/L, mean ± SD	74.75 ± 27.74	1.01 (0.98, 1.03)	0.69
Troponin I, median IQR, ng/mL	0.08 (0.01–0.88)	1.02 (0.95, 1.10)	0.53
Ascending aorta diameter, cm, mean ± SD	4.81 ± 0.72	1.01 (0.39, 2.63)	0.99
Pleural effusion *n* (%)	19 (20.00%)	1.16 (0.22, 6.09)	0.86
Pericardial effusion *n* (%)	23 (24.21%)	0.88 (0.17, 4.59)	0.88
Coronary arteries involved n (%)	6 (6.32%)	2.02 (0.21, 19.53)	0.54
Cross-clamp time, min, mean ± SD	101.85 ± 42.46	1.00 (0.99, 1.02)	0.80
Cardiopulmonary bypass time, min, mean ± SD	189.05 ± 71.77	1.00 (0.99, 1.01)	0.62

*Results are expressed as n (%) or mean ± standard deviation (SD) or median interquartile range (IQR)*.

*BMI, body mass index; TAZ, WW domain–containing transcription regulator protein 1; CI, confidence interval; OR, odds ratio*.

* *P value indicates significance at P < 0.05*.

### The Linear Relationship Between the Preoperative Plasma Level of TAZ and the Postoperative In-Hospital Death in Different Models

**Figure 2:**
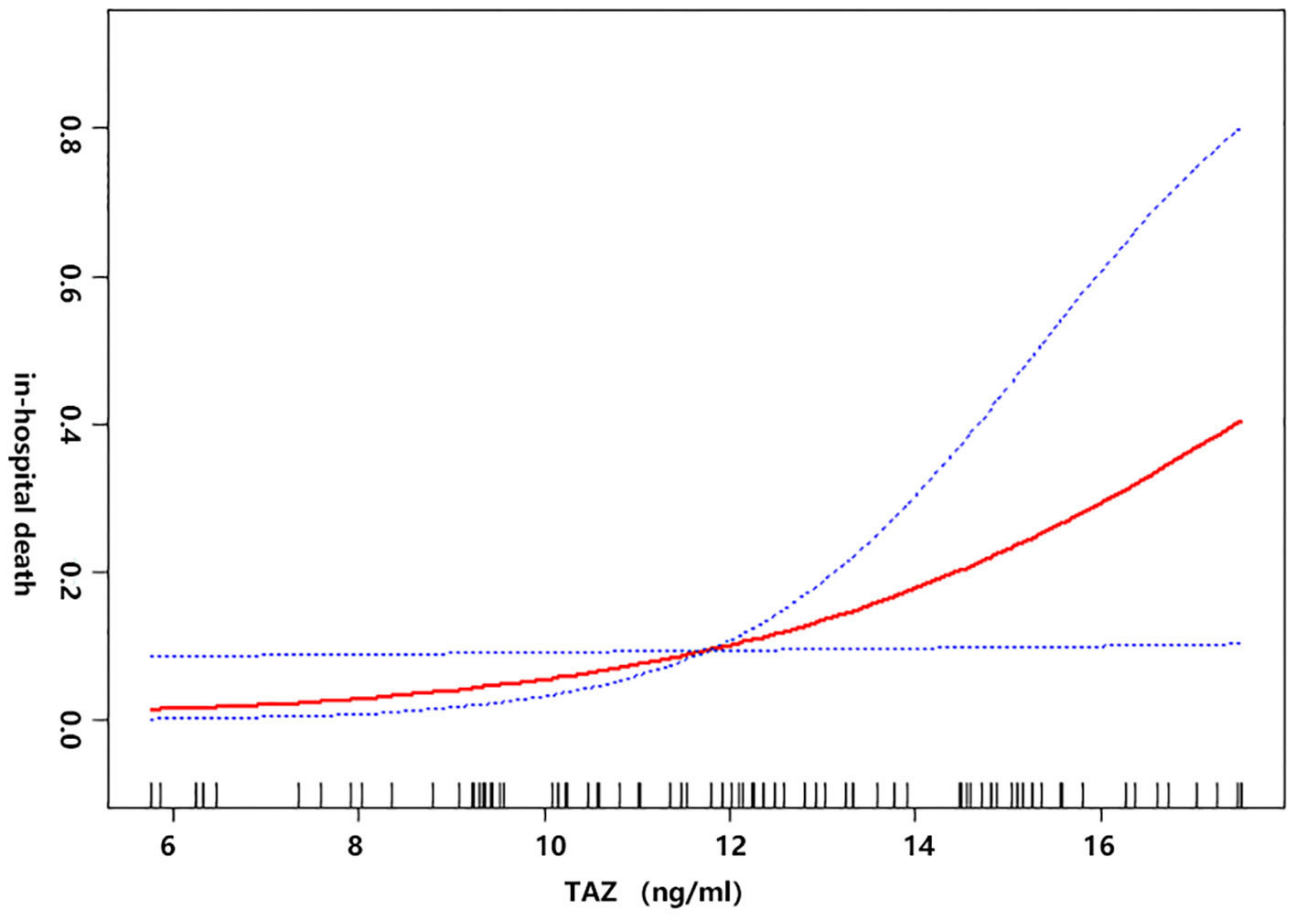
The non-linear relationship between the preoperative plasma level of TAZ and postoperative in-hospital death after adjusting for age, sex, BMI, recent myocardial infarct, serum creatinine (μmol/L) cardiac tamponade, and cardiopulmonary bypass time (min).

Univariate linear regression models were used to evaluate the associations between TAZ expression and postoperative in-hospital death after adjusting for age, sex, BMI, recent myocardial infarct, serum creatinine, cardiac tamponade, and cardiopulmonary bypass time (Figure 2). The red line represents the model's fit spline, while the blue line represents the 95% confidence intervals.

### Multiple Regression Analysis: The Predictive Ability of the Preoperative Plasma Level of TAZ for Postoperative In-Hospital Death

The multiple regression analysis results are shown in Table 3. Three models were constructed: crude model (not adjusted), adjusted model I (adjusted for age, sex, and BMI), and adjusted model II (adjusted for age, sex, BMI, recent myocardial infarct, serum creatinine, cardiac tamponade, and cardiopulmonary bypass time). In the crude model, the preoperative plasma level of TAZ showed a positive correlation with postoperative in-hospital death (OR = 1.33, 95% CI: 1.01–1.74, *P* = 0.04). In adjusted model I and adjusted model II, similar results were indicated (OR = 1.35, 95% CI: 1.01–1.80, *P* = 0.04 and OR = 1.35, 95% CI: 1.01–1.81, *P* = 0.04).

**Table 3:** Relationship between the preoperative plasma level of TAZ and Postoperative in-hospital death in different adjusted models.

**Variables**	**Crude model**		**Adjusted model I**		**Adjusted model II**	
	**OR (95% CI)**	***P*-value**	**OR (95% CI)**	***P*-value**	**OR (95% CI)**	***P*-value**
TAZ (ng/mL)	1.33 (1.01, 1.74)	0.04^*^	1.35(1.01, 1.80)	0.04^*^	1.35 (1.01, 1.81)	0.04^*^
TAZ (ng/mL)						
<12.70	1.0		1.0		1.0	
≥12.70	7.65 (1.49, 39.28)	0.02^*^	8.59 (1.55, 47.65)	0.01^*^	10.08 (1.63, 62.37)	0.01^*^

*Crude model: adjust for none Adjusted model I: adjust for age, sex, BMI Adjusted model II: adjust for age, sex, BMI, recent myocardial infarct, serum creatinine (μmol/L), cardiac tamponade, and cardiopulmonary bypass time (min)*.

*TAZ, WW domain–containing transcription regulator protein 1; CI, confidence interval; OR: odds ratio*.

* *P-value indicates significance at P < 0.05*.

**Figure 3:**
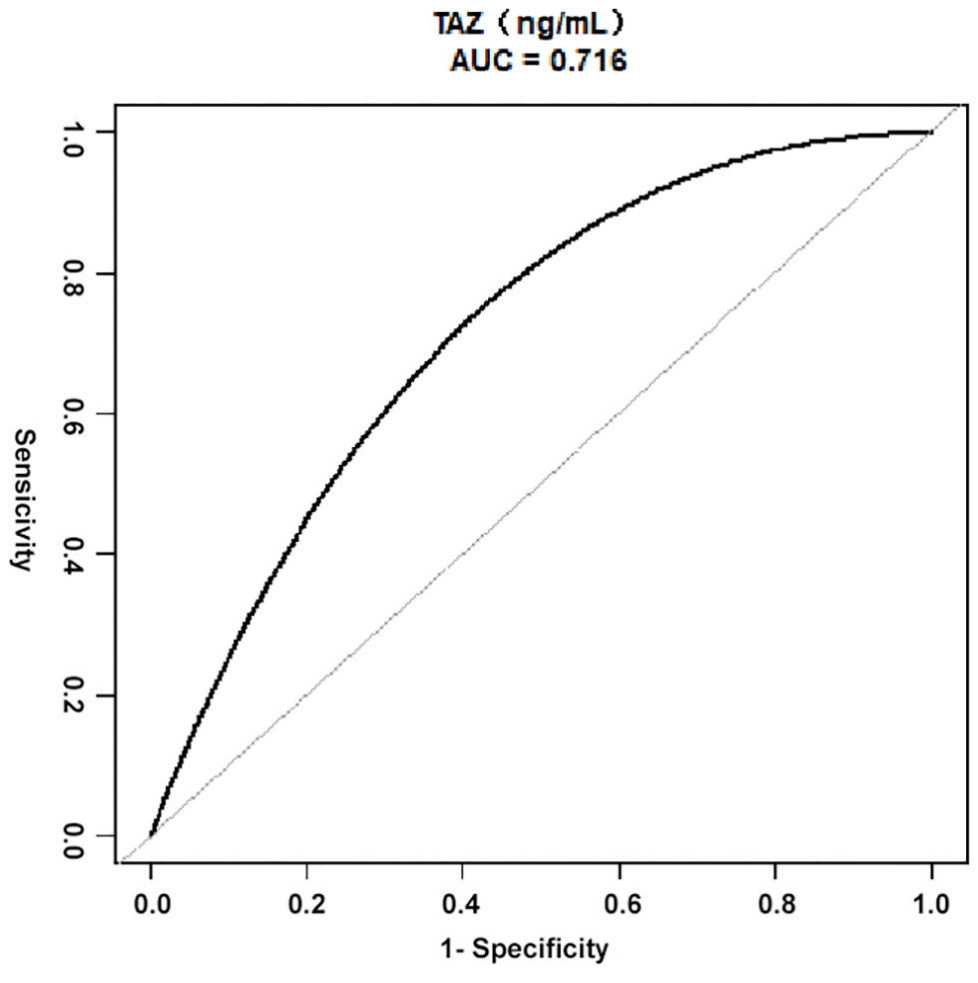
ROC curves for the preoperative plasma level of TAZ in ATAAD, using Bootstrap resampling (times = 500) for the association of postoperative in-hospital mortality. AUC confidence interval and significance tests using Bootstrap resampling. The best threshold was based on maximizing the sum of sensitivity and specificity. The analysis reveals that 12.70 (ng/mL) was the best threshold (AUC: 0.72, 95% CI 0.53–0.86; sensitivity: 68.60%; specificity: 77.78%). ATAAD, Acute type A aortic dissection; ROC, receiver-operating characteristic; AUC, area under the curve; CI, confidence interval; TAZ, WW domain–containing transcription regulator protein 1.

As demonstrated in Figure 3, the ROC curve was used to determine the best threshold of the preoperative plasma level of TAZ by using a bootstrap resampling (times = 500), which showed an area under the curve of 0.72. The best threshold based on maximizing the sum of sensitivity and specificity was 12.70 (ng/mL) (sensitivity: 68.60%; specificity: 77.78%). Multiple regression analysis (Table 3) showed a significant difference between patients stratified by the preoperative plasma level of TAZ = 12.70 (ng/mL). The risk of postoperative in-hospital death in the preoperative plasma level of the TAZ ≥ 12.70 ng/mL group was 7.65 times (OR = 7.65, 95% CI: 1.49–39.28; *P* = 0.02) that of the preoperative plasma level of the TAZ < 12.70 ng/mL group Table 4. After adjustment, the result was still statistically significant: the risk of postoperative in-hospital death in the preoperative plasma level of the TAZ ≥ 12.70 ng/mL group was 10.08 times (OR = 10.08, 95% 1.63–62.37; *P* = 0.02) that of the preoperative plasma level of the TAZ < 12.7 ng/mL group.

**Table 4:** Postoperative outcome for the TAZ < 12.70 ng/ml group and TAZ ≥ 12.70 ng/ml group.

**TAZ (ng/mL)**	** <12.70**	**≥12.70**	***P*-value**
N	61	34	
Postoperative in-hospital death n (%)	2 (3.28%)	7 (20.59%)	0.01^*^

*TAZ, WW domain–containing transcription regulator protein 1*.

* *P value indicates significance at P < 0.05*.

## Discussions

In the history of ATAAD, the mortality of untreated patients with ATAAD is reported to be ~1–2% per hour after occurrence, 50% at the end of the third day, and up to 90% within 30 days (12–15). Medical management for these patients was useless, the postoperative in-hospital mortality rate was over half (57%), and no improvement was observed as the medical technology developed (2). The present guidelines recommend that aortic surgeons try to operate on all patients with ATAAD because 1-month mortality declines almost 70% (3, 4). However, surgeons still need to remain calm and make appropriate, careful surgical choices, even though there has been a significant decline in the in-hospital surgical mortality rate of patients presenting with ATAAD, which has decreased from 25 to 18% in 17 years (2). Some preoperative presentations (such as malperfusion phenomena: neurological or musculoskeletal dysfunction, recent myocardial infarction, and tamponade) have been incorporated into predictive risk models (16, 17) based on the International Registry of Aortic Dissection (IRAD) and individual center data (18, 19). However, the present study reported that recent myocardial infarction, critical preoperative state (including neurological or musculoskeletal dysfunction), and tamponade were not associated with postoperative in-hospital death in this cohort. The reason to the different findings might be that all the small populations of patients received emergent surgery, and the clinical malperfusion phenomena, rather than signs of malperfusion in the CT angiography, did not appear in this early stage. As a result, more easily measurable and precise biomarkers are needed for aortic surgeons to precisely evaluate the postoperative in-hospital mortality of patients and make the best choices for their patients.

TAZ exists in most human tissue, and its functional role is critical in the cardiovascular system (8, 20, 21). Our previous study found that Hippo pathway expression played a key role in hypertrophic cardiomyopathy (22). The central regulating function of the Hippo pathway was identified in a study of the phenotypic switch of vascular smooth muscle cells (VSMC) (21). We also found that clearly disrupted elastic lamellae of variable widths softened the ECM of the ascending aortic wall, possibly inducing Hippo pathway, which promoted the development ATAAD (8). The reduction of Yes-associated protein (YAP) in the middle layer of the aortic wall in ATAAD was confirmed, and its expression was negatively correlated with the ascending aorta diameter, which means that the less YAP in the tissue, the more severe the dissection is (8). It is widely known that TAZ and YAP are transcriptional co-regulators, and they are expressing and functioning similarly (23). YAP shares 45% amino acid identity with TAZ, and both of them play redundant roles in the control of cardiac growth, but the potential functional redundancy of these proteins has not been confirmed. (23). Our study provided an interesting result that TAZ was expressed at a significantly higher plasma level of ATAAD patients. Based on these findings, we propose a hypothesis that the downregulation of YAP expression in the aortic wall might lead to a compensatory increase of its transcriptional coregulator, TAZ, rather than itself, in the blood. If the hypothesis is true, the less YAP in the aortic wall would cause more severe dissection and a higher plasma level of TAZ in ATAAD patients. The increasing TAZ in blood would become a biomarker of the severity of ATAAD.

Thus, this study relied on the clinical data to determine whether there was an association between the preoperative plasma level of TAZ and the severity of ATAAD. The plasma level of TAZ in blood had a significant positive relation to postoperative in-hospital death in all the models. In the crude model, postoperative in-hospital death increased 33% as the preoperative plasma level of TAZ increased 1 ng/ml. In adjusted model I and adjusted model II, the increase in postoperative in-hospital death was 35 and 35%, respectively, when the preoperative plasma level of TAZ experienced an increase of 1 ng/ml. In adjusted model I, basic patient data, such as age, sex, and BMI, were chosen as variables. According to our clinical experience, recent myocardial infarct, serum creatinine, cardiac tamponade, and cardiopulmonary bypass time were added to adjusted model II. After that, we determined that a preoperative plasma level of TAZ = 12.70 ng/ml was a statistically significant cutoff point using the ROC curve. The postoperative in-hospital death of patients with the preoperative plasma level of TAZ ≥ 12.70 ng/ml was 10.08 times that of patients with TAZ < 12.70 ng/ml. This outcome indicates to aortic surgeons that a high preoperative plasma level of TAZ means poor surgical prognosis for ATAAD patients.

Age ≥70 years, previous cardiac surgery, cardiac tamponade, and recent myocardial infarction were reported as independent postoperative in-hospital predictors of mortality (16, 17). However, these factors played less important roles in predicting postoperative in-hospital death in the present study for the following reasons. First, the population of this study (51.01 ± 11.59 years) was younger than that in the International Registry of Acute Aortic Dissection (IRAD, population approximately 60 years old), (16, 17) so we are unable to divide the participants according to an age ≥70 years. Second, the ratio of previous cardiac surgeries in this study (12.79%) was similar to that from IRAD (13.90%) (16). Although there was a significant relationship between previous cardiac surgery and mortality reported in the literature, the results of most subgroups (aortic valve replacement, aortic aneurysm or dissection, and CABG) were not significant in our study. Finally, the predictive effect of malperfusion phenomena on postoperative in-hospital mortality is controversial. The present study reported that malperfusion phenomena, such as recent myocardial infarction, critical preoperative state (including neurological or musculoskeletal dysfunction), and cardiac tamponade, were not associated with postoperative in-hospital death in this cohort. The malperfusion phenomena might appear in the early stage, but all the patients of this study received the surgery urgently.

Several limitations exist in this study. First, this is a retrospective cohort study and provides only weak evidence between exposure and outcome, and it is difficult to distinguish between cause and effect. Second, some patients were diagnosed by the CTA from other hospitals before surgery, and it was difficult to get these CTA to do a detailed analysis, so we used data of clinical malperfusion, such as recent myocardial infarction, critical preoperative state, and cardiac tamponade. Finally, because the study population contains only 95 ATAAD patients who underwent surgery in a very short period (6 month), the results may not be generalizable to all ATAAD patients. Considering the fact that only 9 deaths occurred, the correlations and statistical analyses performed to associate the preoperative plasma level of TAZ with mortality could be improved with a larger prospective study population in the future.

## Conclusions

In conclusion, this study showed for the first time that a high preoperative plasma level of TAZ suggests poor surgical prognosis in ATAAD patients and that patients with the preoperative plasma level of TAZ ≥ 12.70 ng/ml had much higher postoperative in-hospital mortality. As a result, TAZ is related to surgical mortality of patients with ATAAD and merits rigorous study in the future.

## Data Availability Statement

The raw data supporting the conclusions of this article will be made available by the authors, without undue reservation.

## Ethics Statement

The studies involving human participants were reviewed and approved by Ethics Committee of Beijing Anzhen Hospital (Institutional Review Board File 2014019). The patients/participants provided their written informed consent to participate in this study.

## Author Contributions

WJ, YZ, and HZ were expected to have made substantial contributions to the conception. YX and HL helped in sample analysis and contributed to analyzing the results. WJ, YX, and HL play major roles in writing and revising the manuscript. All authors read the manuscript and approved the final version.

## Conflict of Interest

The authors declare that the research was conducted in the absence of any commercial or financial relationships that could be construed as a potential conflict of interest.
